# An Accurate and Robust Method for Spike Sorting Based on Convolutional Neural Networks

**DOI:** 10.3390/brainsci10110835

**Published:** 2020-11-11

**Authors:** Zhaohui Li, Yongtian Wang, Nan Zhang, Xiaoli Li

**Affiliations:** 1 School of Information Science and Engineering, Yanshan University, Qinhuangdao 066004, China; lizhaohui@ysu.edu.cn (Z.L.); yongtian.w@stumail.ysu.edu.cn (Y.W.); znzn@stumail.ysu.edu.cn (N.Z.); 2 Hebei Key Laboratory of Information Transmission and Signal Processing, Yanshan University, Qinhuangdao 066004, China; 3 State Key Laboratory of Cognitive Neuroscience and Learning, Beijing Normal University, Beijing 100875, China

**Keywords:** extracellular recording, spike sorting, deep learning, convolutional neural network

## Abstract

In the fields of neuroscience and biomedical signal processing, spike sorting is a crucial step to extract the information of single neurons from extracellular recordings. In this paper, we propose a novel deep learning approach based on one-dimensional convolutional neural networks (1D-CNNs) to implement accurate and robust spike sorting. The results of the simulated data demonstrated that the clustering accuracy in most datasets was greater than 99%, despite the multiple levels of noise and various degrees of overlapped spikes. Moreover, the proposed method performed significantly better than the state-of-the-art method named “WMsorting” and a deep-learning-based multilayer perceptron (MLP) model. In addition, the experimental data recorded from the primary visual cortex of a macaque monkey were used to evaluate the proposed method in a practical application. It was shown that the method could successfully isolate most spikes of different neurons (ranging from two to five) by training the 1D-CNN model with a small number of manually labeled spikes. Considering the above, the deep learning method proposed in this paper is of great advantage for spike sorting with high accuracy and strong robustness. It lays the foundation for application in more challenging works, such as distinguishing overlapped spikes and the simultaneous sorting of multichannel recordings.

## 1. Introduction

Analyzing the electrophysiological activities of neurons is a basis for exploring brain functions. Neurons in the brain communicate with each other by propagating action potentials, also referred to as “spikes” [1,2,3]. Hence, unraveling the interactions between different neurons is a key step to advancing our understanding of brain science and neural engineering [4]. Prior to interpreting the information carried by the interactions, an essential operation is to separate the spikes of individual neurons picked up by an extracellular electrode [5]. That is, it is necessary to determine which spike corresponds to which neuron, because each electrode records the extracellular field in its vicinity and can detect the spikes emitted by several neurons adjacent to the electrode site [6,7,8,9]. This identification is universally termed spike sorting [10,11,12], which is conducted on the basis of the similarity of spike waveforms. The reason lies in the fact that each neuron fires spikes of a particular shape, depending on the morphology of its dendritic tree, its distance and orientation relative to the recording site, etc. [11,13].

In fact, there are usually four main steps in conventional approaches for spike sorting: (1) Bandpass-filtering (e.g., 300–3000 Hz) the recorded raw data to eliminate the interferences of high-frequency noise and low-frequency field potential; (2) detecting the spikes by determining an amplitude threshold [14] or utilizing other improved methods [15], such as wavelet transforms [16] and fuzzy decision [2]; (3) extracting discriminative features from the detected spikes, frequently using approaches such as principal component analysis (PCA) [17,18,19] and wavelet transform coefficients [14,20,21]; (4) grouping the points in feature space to obtain clusters associated with individual neurons. Many classical and advanced methods have been adopted for this purpose, such as superparamagnetic clustering (SPC) [14], k-means clustering [22], and a mixture of Gaussians [23,24].

In this paper, we focus on a clustering algorithm, i.e., how to correctly isolate spikes originating from different neurons. Compared with semi-automatic methods [25,26], a fully automated process [27,28] requires no human input, consequently reducing time consumption and subjective bias. However, it sacrifices accuracy because it may ignore the distribution of clusters when a subset of a cluster exceeds the implicit boundary [29]. Thus, it is indispensable to manually resort falsely classified spikes, which is time-consuming and labor-intensive. Moreover, with the development of modern microelectronics and electrophysiology, high-density microelectrode arrays containing hundreds of channels have been widely applied in neuronal population recordings [3,30,31,32]. As a result, it is possible to simultaneously record the firing activities of hundreds or even thousands of neurons [33], involving tremendous amounts of data. Thus, current methods require substantial manual intervention, especially in long-term experiments, which makes them slow and unreliable. Inevitably, false classifications affect the results in subsequent analysis. Therefore, it is urgent to develop an effective method to reduce the workload of manual classification while ensuring accuracy and robustness [34]. Here, in light of recent advances in deep learning techniques [1,35], we took the manually classified spikes in an initial interval of the experimental data as the “ground-truth” labels, which were used to train a convolutional neural network (CNN) [36]. Then, this CNN could be used to automatically cluster the remaining data, thereby drastically reducing the time needed for spike sorting with high accuracy.

As an important type of deep learning technique, the CNN has been successfully used in computer vision since the early 21st century [36]. It performs well not only in object detection and brain image analysis [37,38], but also in the medical research field [39,40,41]. In recent years, deep learning techniques have also been used in spike detection and spike sorting. Yang et al. introduced a deep learning algorithm, named the principal component analysis network (PCANet), to reduce the computational complexity of conventional spike sorting algorithms [42]. However, the method did not show better sorting performance on overlapped spikes. The SpikeDeeptector method employed a CNN to select meaningful channels for brain–computer interfaces [15]. It considered a batch of waveforms to construct a single feature vector, which were fed to a deep learning method. Unlike SpikeDeeptector, we used a CNN to distinguish the categories of spikes in this paper. Both simulated data and experimental data were employed to evaluate the performance of our proposed model. By using the “Wave_Clus” database [43], we compared our proposed one-dimensional (1D)-CNN model with a recently developed method named “WMsorting” [44] and a deep-learning-based multilayer perceptron (MLP) model [45]. “WMsorting” proposed a spike feature extraction method using wavelet packet decomposition and mutual information. It is a semi-supervised solution for the automation of a spike sorting framework. The MLP approach constituted a deep-learning-based spike sorting method on extracellular recordings from a single electrode, improving on PCA + K-means clustering. We also compared our 1D-CNN model to some traditional methods, such as a PCA-based feature extraction with fuzzy C-means (FCM) clustering, a correlation coefficient-based (CORR) method with fuzzy clustering, first and second derivative extrema (FSDE) of spike data combined with k-means clustering, and a fusion feature strategy along with a support vector machine (SVM). Furthermore, we used the experimental data recorded from the primary visual cortex of a macaque monkey to test our 1D-CNN model and “WMsorting.”

## 2. Materials and Methods

### 2.1. Architecture of the 1D-CNN Model

**Figure 1:**
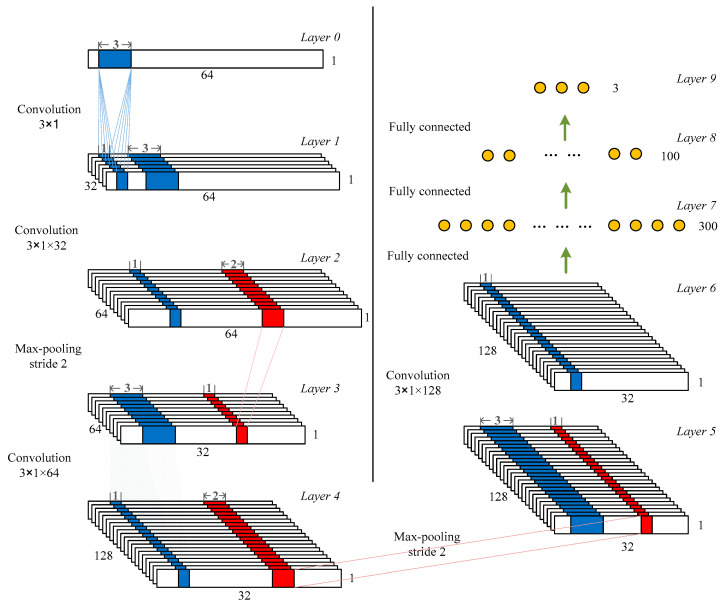
Architecture of the proposed convolutional neural network (CNN) model used in the simulated database. The blue region corresponds to convolutional processes, and the red region represents the pooling. The stride of all convolutional layers was 1.

The standard architecture involved in a CNN includes the convolutional layer, rectified linear activation function, pooling layer, and fully connected layer [36]. Figure 1 illustrates the proposed CNN architecture working on a simulated database. The model contained four convolutional layers and two pooling layers, followed by a fully connected neural network and a softmax classifier. The CNN model used for the experimental data had a similar architecture. The only difference was the length of the input layer, whereby the former had a value of 64 and the latter had a value of 12. The details of the model are described as below.

**Convolutional layer:** The main building block of a CNN is the convolutional layer, which completes most of the computationally intensive lifting and extracts features from the input signals. In this paper, four convolutional layers were arranged into nine feature maps [36]. The original intention for this choice was because, when the number of convolutional layers was greater than four, the accuracy nearly stopped growing. For all convolutional layers, the stride was 1, the kernel size was 3, and the padding was the same. If the stride is too small, the extracted features are more comprehensive and do not miss too much information. Due to that, the stride was set to 1. Compared with using a larger convolution kernel, using smaller convolution kernels can obtain more feature information with a smaller amount of calculation. Thus, the kernel size of the convolutional layer was set to 3. We filled the edges of the input matrix with zero padding, allowing us to filter the edges of the input matrix. One of the advantages of zero padding was that it allowed us to control the size of the feature map.

**Rectified linear activation function and regularization techniques:** The rectified linear unit (Relu) layer is used for addressing the problem of optimization by mapping nonlinearity into the data [36]. The reason we chose Relu was that it could be used to alleviate the problem of gradient disappearance. It was designed as an activation function for layers 1, 2, 4, 6, 7, and 8. The softmax function was employed in layer 9 (last layer of the network). Using the softmax classifier, the prediction of a cluster to which the input data belong can be realized. The batch normalization, which kept the inputs closer to a normal distribution, was applied to the output of the convolutional layer. The dropout [46], another regularization technique, was applied to reduce overfitting by randomly setting the values of some input neurons to zero before the fully connected layers.

**Pooling layer:** The max pooling layer is implemented to reduce the computational complexity of conventional sorting algorithms. Max pooling compares every pair of numbers and outputs the bigger value. In this paper, two max pooling layers were used with a kernel size of 3. The stride was set to 1, and the filter convolved around different layers of the feature map by sliding one unit each time.

**Fully connected layer:** The output dimensions of the final connected layer depend on the number of classes. For an *n*-class (*n* kinds of spikes) problem, the output dimensions were set to *n*. There were three kinds of spikes in the simulated data, that is, the number of output dimensions was three. In the experimental data, we separately set-up the output dimensions for each channel, depending on the number of clusters determined by the spike sorting algorithm and human intervention.

As shown in Figure 1, the input layer (layer 0) with a resolution of 64 × 1 was convolved with 32 filters of kernel size 3 to form the first layer (layer 1). The second convolutional layer with a kernel size of 3 (64 filters) was applied to produce the second layer. A max pooling layer was employed with a kernel size of 2 (layer 3). A convolutional operation was administered on layer 3 (32 × 64) to form layer 4. The feature maps from layer 4 were once again pooled with a kernel size of 2 to construct the last max pooling operation (layer 5). Then, another round of convolution was employed with a kernel size of 3. The next three layers were the fully connected layers. The neurons of feature maps in layer 6 were fully connected to 300 neurons in layer 7. Layer 7 and layer 8 were respectively fully connected to 100 and *n* outputs in layers 8 and 9.

### 2.2. Simulated Database and Experimental Datasets

The simulated database created in “Wave_Clus” [14] has been widely used in the evaluation of several spike sorting algorithms [42,44,47]. In total, 594 different average spike waveforms compiled from real recordings were adopted as templates to construct simulated signals. Randomly selected spikes were superimposed at random times and amplitudes to mimic the background noise. Then, it was feasible to realize different signal-to-noise ratios (SNRs) by altering the ratio between the amplitudes of the noise and signals. More details related to the generation of the simulated database can be found in [43].

**Figure 2:**
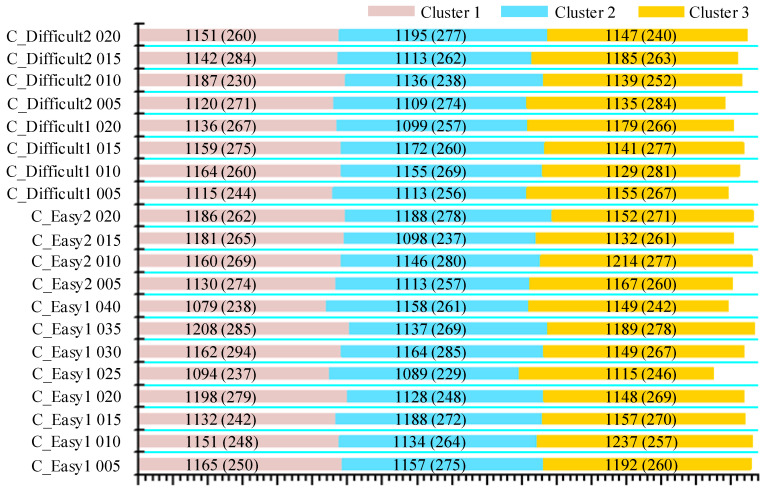
Details of the simulated database. Different colors represent different clusters. The number in parentheses represents the number of overlapped spikes.

There were four big datasets used in our experiments, i.e., C_Easy1, C_Easy2, C_Difficult1, and C_Difficult2. C_Easy1 contained eight noise levels ranging from 0.05 to 0.4 with a step of 0.05. The other three datasets contained four levels ranging from 0.05 to 0.2. “Easy” and “difficult” were set to characterize the overlapping degrees between spikes. Spikes of 64 sampling points were extracted from the continuous data by using the ground-truth spike times denoted in each dataset. Obviously, these spikes could be treated as labeled samples, making them suitable for testing our supervised deep learning method. The sampling rate of the simulated database was 24 kHz. Detailed information of the database is provided in Figure 2.

**Figure 3:**
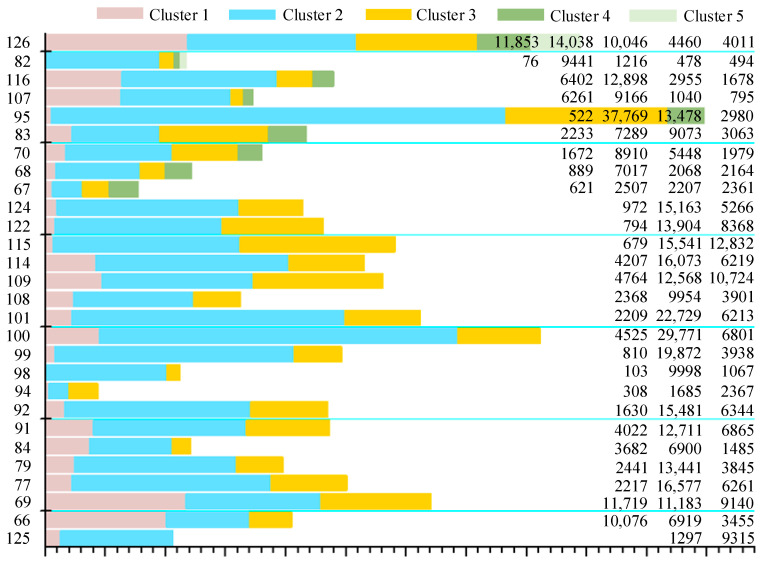
Details of the experimental datasets. Different colors represent different clusters. The number in parentheses represents the number of overlapped spikes.

The experimental datasets were obtained from public databases (Collaborative Research in Computational Neuroscience, CRCNS) [48,49]. The data were recorded from the primary visual cortex (V1) of a macaque monkey with 64-site multi-electrode arrays (eight penetrations, eight sites per penetration). The number of electrode contacts ranged from 65 to 128. After the spikes were isolated via superparamagnetic clustering, manual resorting was conducted to avoid possible errors. The sampling rate of the experimental datasets was 24.4 kHz. The dataset provided spikes of single neurons, including their waveforms and firing times. Here, we removed the channels with very few spikes in at least one cluster (the number of spikes was less than 0.5% of the total), because there were not enough labeled samples to train the 1D-CNN. Figure 3 lists the remaining channels with detailed cluster information, which were used to test the performance of our deep learning method.

### 2.3. Training and Testing

**Figure 4:**
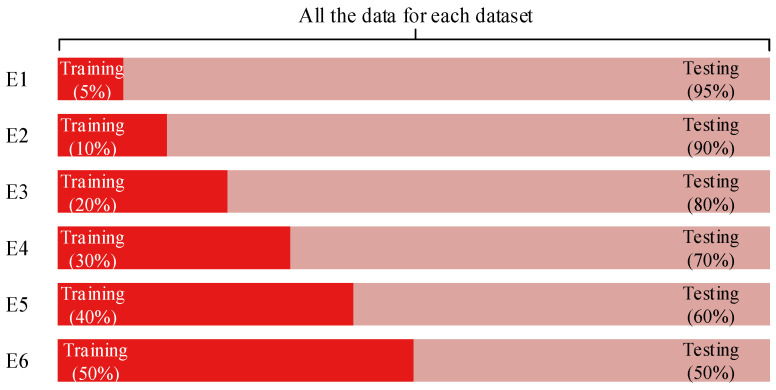
Proportion of data in training set and testing set for the six experiments. E = experiment.

Six experiments were performed for each dataset. The proportion of data used for the training and testing procedures is illustrated in Figure 4. For each dataset, the training set separately held 5%, 10%, 20%, 30%, 40%, and 50% of the total data, and the corresponding testing set held the remaining 95%, 90%, 80%, 70%, 60%, and 50%, respectively. We separately used 50% of the total data in C_E1_015 and C_D1_015 as the training set and the remaining data as the validation set to adjust and evaluate our model. When our model was determined, other datasets were used to evaluate the performance, and they were divided into a training set and testing set.

To evaluate the performance of the model, accuracy, which was calculated as the percentage of correctly classified samples over all data, was utilized to compute the score of the entire classification, and it was used in the analysis of experimental data. In addition, considering the prevalent imbalance of data distribution in the experimental data, the macro-average F-measure (Macro_F) was also employed to describe the classification effects. Macro_F is more sensitive to the classification quality, and it is defined as follows [50]:

(1) $$Macro\_F=\frac{1}{N}\overset{N}{\underset{i=1}{\sum }}\frac{2\times P_{i}\times R_{i}}{P_{i}+R_{i}}$$

where $P_{i}=TP_{i}/TP_{i}+FP_{i}\;$ is the recall, $R_{i}=TP_{i}/TP_{i}+FN_{i}$ is the precision, and *N* is the number of clusters. TP denotes true positives, FP denotes false positives, and FN denotes false negatives.

## 3. Results

### 3.1. Simulated Database

In this section, the classification accuracy of the proposed 1D-CNN model is compared with “WMsorting”, the deep-learning-based MLP model, and four traditional methods using the “Wave_Clus” database. The four traditional methods utilized PCA-based and correlation-based (CORR) feature extraction with FCM as the clustering method and two other public methods (fusion + SVM and FSDE + K-means). Details of these traditional methods can be found in [44]. The results for the data with different noise levels are shown in Table 1 and Table 2.

Although “WMsorting” showed relatively high accuracy on the C_Easy1, C_Easy2, and C_Difficult2 datasets, it was not robust. The accuracy of “WMsorting” on C_Difficult1_020 was 85.38% and 86.45% when the feature dimensions were 3 and 10, respectively, which were much lower than the results of other datasets. The feature dimension is an important parameter in sorting evaluation, and it is determined with reference to the best Micro_F (micro-average F-measure) [51] score of the PCA-based method. On the other hand, the accuracy of our proposed deep learning method on C_Difficult1_020 was above 98% except for experiment 1, whose accuracy was 95.16%. That is, even when we used only 5% of the spikes to train the 1D-CNN, we still obtained an accuracy improvement of 8.71%. In fact, when the number of labeled spikes increased, i.e., in the other five experiments, our results could enhance the clustering accuracy by more than 10% compared to “WMsorting.” In the other datasets, although the enhancement was not so significant, our deep learning method behaved better than “WMsorting.” Additionally, the improved accuracy is shown in the last column of Table 1, indicating that the best accuracy of our proposed model was relatively higher than the best accuracy of “WMsorting” on all datasets.

Compared to traditional methods, our proposed deep learning method had more obvious advantages. Traditional methods worsened with increasing noise level for all datasets, with an error rate as high as 46%, while our error rate was around 2%. For lower noise levels, our accuracy was still better than that of traditional methods.

The deep-learning-based MLP model used 10% of the data nearest to the cluster centers as the training data; thus, we used experiment 2 (10% of the total data as training set) in the last column of Table 2. As can be seen, the deep-learning-based MLP model did not perform well with a higher noise level, especially on the C_Difficult2_020 or C_Difficult2_015 datasets. The deep-learning-based MLP model’s accuracy on C_Difficult2_020 was 51.55%, which was 42.8% lower than ours.

As the number of spikes used in the training set gradually increased (from 5% to 50% of the total), the accuracy exhibited a slight tendency to increase (0.607% on average). It was also shown that 5% (170 spikes) of the data were enough to train the proposed 1D-CNN model in most cases. On the other hand, with the increase in noise level (from 5% to 20%), the accuracy had no significant reduction. Both factors confirmed that our proposed deep learning method is robust.

**Figure 5:**
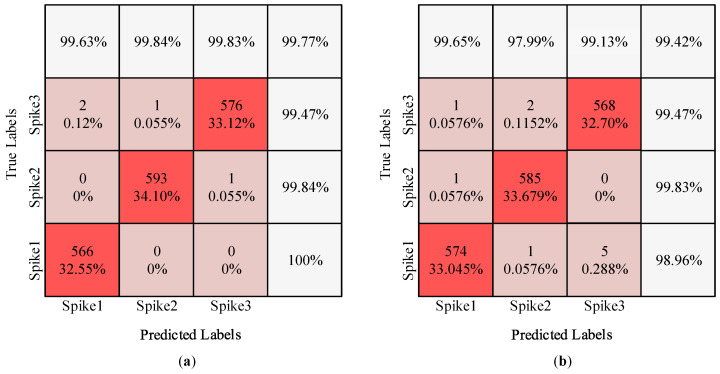
Confusion matrix: (**a**) C_Easy1_noise015; (**b**) C_Difficult1_noise015.

Furthermore, we took experiment 6 for two datasets (C_Easy1_noise015 and C_Difficult1_noise015) as an example to illustrate the results in more detail. First, we observed the accuracy of individual classes by constructing confusion matrices, as shown in Figure 5a,b. Each row in the confusion matrix represents a true label, while each column represents a predicted label. The bold percentages denote the proportion of correct true labels for each cluster. Clearly, there was only a small difference between the accuracy of individual classes and the overall accuracy, which is consistent with the balanced spike distribution of each cluster in the “Wave_Clus” database. That is, the proposed 1D-CNN model performed excellently for different clusters, indicating that it is an accurate and robust method for spike sorting.

**Figure 6:**
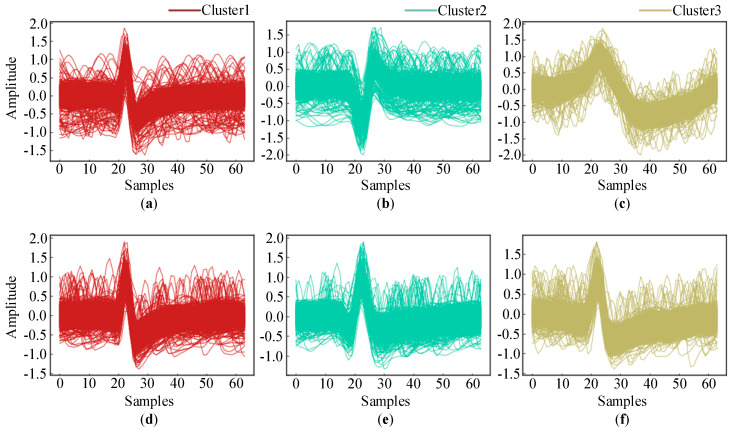
Waveforms of spikes in each cluster: (**a**–**c**) C_Easy1_noise015; (**d**–**f**) C_Difficult1_noise015.

Moreover, the accuracy for the C_Difficult1_noise015 dataset was clearly lower than that for C_Easy1_noise015. In order to explain the reason behind this, we plotted the correctly clustered spikes in Figure 6 and the falsely classified spikes in Figure 6. Obviously, the spike waveform shapes of the three clusters in C_Easy1_noise015 were quite different and relatively easy to distinguish. On the other hand, the spike waveform shapes in C_Difficult1_noise015 were similar to each other and difficult to distinguish.

**Figure 7:**
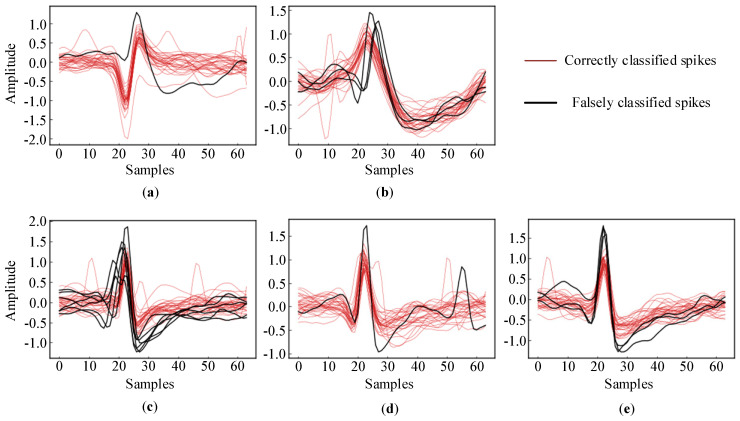
Falsely classified spikes and randomly selected correctly classified spikes: (**a**,**b**) C_Easy1_noise015; (**c**–**e**) C_Difficult1_noise015.

Figure 7 shows all the falsely classified spikes and a small subset of the correctly classified spikes in the two datasets. As illustrated in Figure 7a,b, only one spike in “Cluster 2” and three spikes in “Cluster 3” in C_Easy1_noise015 were falsely classified due to their very similar shapes. The spikes in “Cluster 1” in C_Easy1_noise015 were all correctly classified. On the other hand, there were more falsely classified spikes in C_Difficult1_015. Clearly, they were severely distorted by the noise and had similar shapes, which hindered their sorting into the corresponding clusters. However, compared to the accuracy of 91.18% obtained using the “WMsorting” method, our proposed deep learning approach shows a significant improvement with an accuracy of 99.42%.

**Figure 8:**
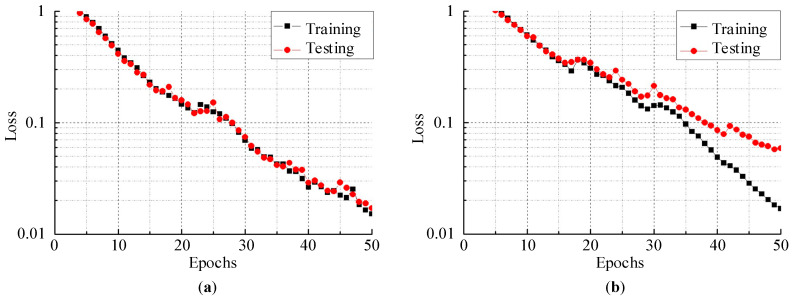
The cross-entropy loss function on the training set and testing set: (**a**) C_Easy1_015; (**b**) C_Difficult1_015.

In addition to clustering accuracy, the loss function is an evaluation index for the performance of deep learning methods. Cross-entropy is a frequently used function that measures the degree of difference between the actual outputs and expected outputs of a CNN model. In other words, a smaller cross-entropy loss function denotes the two outputs being closer. On the other hand, the loss function helps determine whether a model is overfitting or underfitting the data by comparing the training loss and testing loss. The training and testing cross-entropy losses on both datasets (experiment 6 for C_Easy1_noise015 and C_Difficult1_noise015) are plotted in Figure 8. In our proposed model, the training epoch was set to 50. Obviously, although there were occasional rises, the training loss and testing loss decreased along with the increase in the number of epochs. The testing loss of C_Easy1_015 decreased to 0.01708 at the 50th epoch, and the testing loss of C_Difficult1_015 was 0.05861 at the 50th epoch. These values were small enough to demonstrate the robustness of our proposed model. Furthermore, the small difference between the training loss and testing loss showed that our model neither overfitted nor underfitted the data.

### 3.2. Experimental Datasets

**Figure 9:**
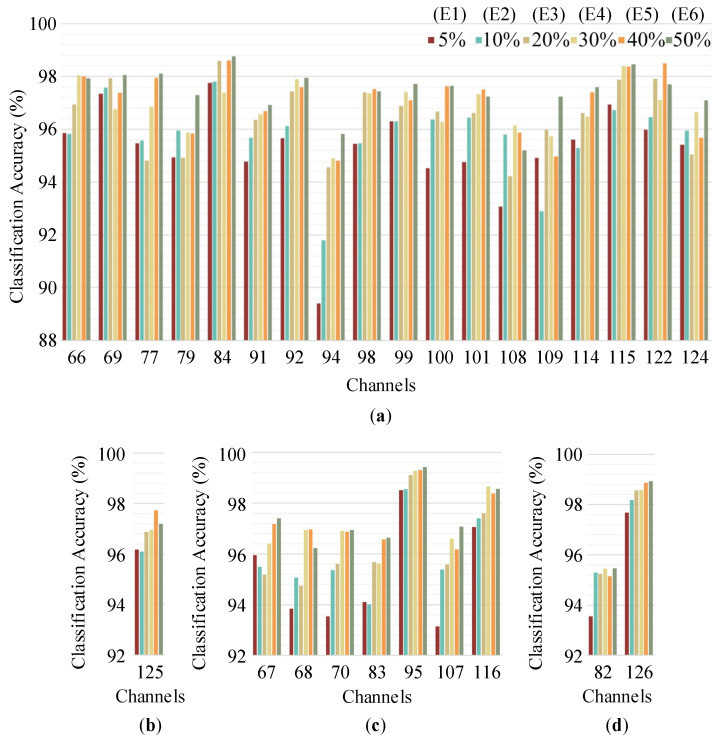
Classification accuracies of the recording channels with different numbers of spike clusters: (**a**) Three clusters; (**b**) two clusters; (**c**) four clusters; (**d**) five clusters.

Similarly, six experiments with different proportions of training data were performed on the extracellular recording spikes to further demonstrate the effectiveness of our method. In Figure 9, the classification accuracies are presented according to the number of spike clusters (ranging from two to five). It can be seen that most of the results achieved accuracies higher than 96.5% regardless of the number of labels used to train the 1D-CNN. In fact, the average accuracy of all experiments was 96.53%, which is an acceptable level. However, it should also be noted that the accuracies of some channels were significantly lower than those of others. For example, in the 94th channel, the classification accuracy of experiment 1 (5% of the data used as the training set) was 89.40%. The reason for this is that the number of spikes in the training set was 218, which was not enough to effectively train the 1D-CNN model. When the number of training spikes increased, the classification accuracy improved, e.g., it was 91.80% in experiment 2 (10% of the data used as the training set) and 94.60% in experiment 3 (20% of the data used as the training set).

The above accuracies of each dataset were calculated as a function of the total spikes. As is known, there is always an imbalanced data distribution in experimental datasets; thus, Macro_F is an appropriate criterion to evaluate the classification quality. The results of this evaluation are given in Table 3. Taking the 98th channel as an example, the accuracy in experiment 1 was 95.44%, but the corresponding Macro_F score was 74.66%. Clearly, there was a great difference. The reason is that the minimum number of spikes in the three clusters was 103 and the corresponding number of spikes involved in the training set in experiment 1 was only 21. Obviously, this was not enough to adequately train the 1D-CNN to achieve a desirable output. It should also be noted that the Macro_F score increased to >90% when the number of spikes in the training set was >30% of the total number of spikes (i.e., experiments 4, 5, and 6). In fact, most Macro_F scores were above 95% in experiment 6. In the 84th and 116th channels, scores of 99% were achieved. Thus, as long as there are enough spikes for training the proposed 1D-CNN, it is very likely to obtain a close approximation of the ideal spike sorting results.

The experimental data were also analyzed using “WMsorting”, and the Macro_F scores are shown in Table 3. We separately carried out two experiments using “WMsorting” with three and ten feature dimensions. We used the best obtained accuracy in each channel from our 1D_CNN model and “WMsorting” to calculate the improvement. As can be seen, our 1D_CNN model provided a better result.

## 4. Discussion

In this paper, we designed a 1D-CNN model to improve the accuracy and robustness of spike sorting. The novel deep learning method exhibited a high sorting accuracy in the simulated database, even when the data suffered from severe noise. In the experimental datasets, our proposed model still performed well in classifying the spikes recorded by the extracellular electrodes in the primary visual cortex of a macaque monkey. Thus, it is reasonable to conclude that our proposed model showed a good performance in terms of accuracy and robustness.

There were two factors influencing the performance of the method, i.e., sampling points and labels. First, the number of sampling points in each spike had a significant impact on the accuracy. As shown in Table 1, the accuracies of C_Easy1_015 and C_Difficult1_015 in experiment 6 are 99.77% and 99.42%, respectively. However, when we downsampled the spike waveforms from 64 points to 16 points, the corresponding accuracies were reduced to 99.14% and 87.91%. Clearly, the reduction seen for the C_Difficult1_015 dataset was much greater than that seen for C_Easy1_015. This is because the spike waveforms of different clusters in C_Difficult1_015 were more similar to each other. The lower accuracies of the experimental datasets with 12 sampling points also verified this effect. That is, with fewer spike sampling points, it was more difficult for 1D-CNN to cluster the data correctly. A possible reason is that the 1D-CNN could not “learn” enough features from the limited spike sampling points. Secondly, as with other deep learning approaches [15,52,53], the number of labels in the training set was of great importance to the clustering accuracy. From the previous analysis of simulated data, it was found that the accuracy could reach over 99.5% when there were more than 60 training spikes of each cluster available in the “easy” datasets. Moreover, the 1D-CNN needed at least 120 spikes of each cluster, with over 98.4% accuracy guaranteed in the “difficult” datasets. As far as the real data were concerned, considering fewer sampling points and severe noise, there should be more than 200 training spikes of each cluster to obtain an accuracy of 95%. Thus, there is no doubt that the accuracy increases with the number of training spikes. When considering both factors, a sufficient number of spikes with enough sampling points in the training set can reliably guarantee a high accuracy and strong robustness. On the other hand, it is time-consuming and laborious to obtain a large number of training spikes. Therefore, it will be necessary to find a tradeoff between performance and cost in real applications.

Although “WMsorting” is a semi-supervised algorithm [54], whereas the method presented here is fully supervised, the degree of manual interventions was almost the same. There were about 170 spikes in the training set for experiment 1 using our proposed method and 180 spikes using “WMsorting.” Hence, the workload when using our method with respect to labeling is equivalent to that when using a semi-supervised algorithm. Meanwhile, the accuracy of our proposed deep learning method was improved with respect to “WMsorting,” especially for the “difficult” datasets.

Compared to traditional methods and the deep-learning-based MLP model [45], our proposed 1D-CNN model had more obvious advantages on robustness. With the increase in noise level for all datasets, traditional methods worsened with an error rate as high as 46.28%, and the deep-learning-based MLP model worsened with an error rate as high as 48.45%. Our error rate was around 2% for all datasets, which was more robust. 

We used the Anaconda software (4.4.10, Anaconda Company, Austin, USA) and implemented the proposed CNN architecture using Keras in Python 3. When performed on a dataset with 1738 training spikes and 1739 testing spikes using an Intel(R) Core(TM) i7-7820X central processing unit (CPU) equipped with a Nvidia GeForce RTX 2070 graphics processing unit (GPU) and 16 GB random-access memory (RAM), the model took approximately 17 s for training and 2 s for testing. This means that our proposed deep learning method could classify a spike in one millisecond. If the performance of the hardware were further improved, the computation time would be shorter. Thus, it is feasible to apply our 1D-CNN method in online spike sorting.

## 5. Conclusions

We proposed a novel deep learning method based on a 1D-CNN, which can be used in online or offline automatic spike sorting. In future works, we will improve this CNN model to effectively cluster overlapping spikes and expand it to simultaneously classify multichannel recordings. As is well known, the shapes of overlapping spikes can appear with various and complicated changes, whereby the superposition time and the unit waveform are always different. This complicates spike sorting. In addition, high-density multielectrode arrays have been used to record the firing activities of large ensembles of neurons. Thus, a spike waveform may be recorded by multiple electrodes at the same time. How to distinguish spikes simultaneously recorded by different electrodes then becomes another important problem to be solved in spike sorting.

## Figures and Tables

**Table 1:** Comparison of classification accuracy on simulated database (using “WMsorting”). 1D, one-dimensional.

Dataset	NL ^1^	WMsorting		1D-CNN						Improvement
		F ^2^ = 3	F = 10	E1 ^3^	E2	E3	E4	E5	E6	
C_Easy1	005	99.60	99.52	99.64	99.53	99.40	99.63	99.67	99.77	0.17
	010	99.66	99.57	99.52	99.72	99.82	99.88	99.86	99.94	0.28
	015	99.71	99.63	99.61	99.49	99.82	99.84	99.81	99.77	0.13
	020	99.57	99.48	99.33	99.33	99.64	99.84	99.76	99.88	0.31
	025	99.45	99.42	99.55	99.56	99.81	99.74	99.85	99.70	0.40
	030	99.57	99.48	99.49	99.65	99.71	99.18	99.62	99.71	0.14
	035	99.43	99.38	99.49	99.43	99.65	99.56	99.20	99.60	0.22
	040	99.76	99.70	99.35	99.51	99.56	99.79	99.75	99.82	0.06
C_Easy2	005	99.50	99.41	99.72	99.64	99.71	99.66	99.66	99.88	0.38
	010	99.52	99.40	99.58	99.62	99.75	99.76	99.91	99.83	0.39
	015	99.44	99.38	99.32	98.89	99.23	99.33	99.61	99.77	0.33
	020	99.29	99.35	99.19	99.69	99.33	99.55	99.62	99.66	0.31
C_Difficult1	005	94.47	95.00	98.07	99.21	99.11	99.11	98.97	99.05	4.21
	010	92.89	93.76	98.99	99.00	99.09	99.46	99.66	99.83	6.07
	015	90.18	91.18	97.76	98.43	98.63	99.18	98.75	99.42	8.24
	020	85.38	86.45	95.16	98.54	99.01	98.83	98.73	98.83	12.38
C_Difficult2	005	99.23	99.44	99.22	98.88	99.48	99.66	99.85	99.82	0.41
	010	98.93	99.51	99.48	99.78	99.78	99.84	99.99	99.94	0.48
	015	98.05	99.51	99.57	99.58	99.53	99.67	99.71	99.71	0.20
	020	95.99	99.66	99.58	99.75	99.61	99.75	99.67	99.83	0.17

^1^ Noise level; ^2^ feature dimensions; ^3^ experiment.

**Table 2:** Comparison of classification accuracy on simulated database (using traditional methods and deep-learning-based multilayer perceptron (MLP) model). PCA, principal component analysis; FCM, fuzzy C-means; FSDE, first and second derivative extrema; CORR, correlation-based; SVM, support vector machine.

Dataset	NL ^1^	PCA + FCM		FSDE + K-means	CORR + FCM		Fusion + SVM	MLP	1D-CNN
		F ^2^ = 3	F = 10	F = 3	F = 3	F = 10	F = 10		E2 ^3^
C_Easy1	005	99.37	99.35	94.62	97.50	97.38	98.66	99.26	99.53
	010	99.72	99.72	95.54	94.04	96.45	98.98	99.43	99.72
	015	99.25	99.28	94.45	90.54	94.94	98.22	99.25	99.49
	020	99.40	99.40	95.08	88.77	92.43	97.35	99.19	99.33
	025	99.24	99.24		84.41	86.84	95.45		99.56
	030	98.73	98.59		81.50	80.83	88.66		99.65
	035	97.76	95.16		77.02	73.80	83.22		99.43
	040	96.49	68.54		75.58	64.62	78.12		99.51
C_Easy2	005	98.48	98.68	94.81	93.20	96.04	92.23	98.68	99.64
	010	97.16	98.24	94.83	86.02	82.19	92.93	98.49	99.62
	015	92.52	94.49	94.96	83.05	82.82	89.80	97.19	98.89
	020	85.20	88.60	92.71	79.81	78.22	86.24	95.20	99.69
C_Difficult1	005	95.86	72.54	94.50	83.48	86.08	97.58	98.78	99.21
	010	89.56	66.11	94.78	65.69	71.55	94.81	98.93	99.00
	015	76.41	61.33	93.81	57.49	58.84	87.85	97.55	98.43
	020	63.03	54.05	90.60	53.72	53.81	78.59	96.62	98.54
C_Difficult2	005	98.69	98.81	94.38	91.50	94.50	87.40	98.49	98.88
	010	98.64	98.76	94.48	90.96	96.33	88.07	94.66	99.78
	015	94.39	97.33	87.18	88.17	96.02	74.65	82.20	99.58
	020	84.63	83.37	81.71	84.77	95.48	67.25	51.55	99.75

^1^ Noise level; ^2^ feature dimensions; ^3^ experiment.

**Table 3:** Macro_F scores on experimental datasets. 1D, one-dimensional.

Channels	Number of Clusters	WMsorting		1D_CNN						Improvement
		F ^1^ = 3	F = 10	E1 ^2^	E2	E3	E4	E5	E6	
125	2	88.94	86.21	87.33	90.96	94.49	97.00	94.49	97.50	8.56
66	3	94.56	95.36	95.16	96.33	96.84	97.34	97.67	98.17	2.81
69	3	96.43	96.49	97.33	97.34	97.66	96.34	97.15	98.17	1.68
77	3	84.05	87.84	89.45	91.78	91.67	96.33	95.97	97.33	9.49
79	3	91.59	92.28	91.49	95.49	94.99	96.35	96.33	96.16	3.88
84	3	92.26	94.02	96.51	98.17	98.00	98.00	97.67	99.00	4.98
91	3	94.37	95.29	94.33	96.00	95.32	95.32	95.32	96.30	1.01
92	3	92.31	94.38	93.33	95.51	94.63	95.65	95.51	95.65	1.27
94	3	87.29	88.61	88.50	88.11	93.01	92.14	93.50	94.36	5.75
98	3	66.18	72.73	74.66	80.06	79.33	90.25	90.00	90.14	17.41
99	3	83.29	87.16	81.69	90.58	91.74	92.09	90.35	93.29	6.13
100	3	89.92	90.64	88.31	93.38	96.00	94.98	96.17	97.66	7.02
101	3	84.28	85.62	87.34	90.78	94.01	94.00	93.80	95.85	10.23
108	3	90.29	90.61	93.30	94.34	94.65	95.67	94.87	95.50	4.89
109	3	90.29	92.52	94.18	94.00	95.83	94.01	95.00	97.49	4.97
114	3	92.49	91.06	91.82	92.73	93.43	95.66	96.99	96.32	3.83
115	3	89.90	88.61	91.33	92.78	94.44	94.62	95.63	96.14	6.24
122	3	86.11	90.60	90.35	93.30	94.44	96.84	95.97	96.01	5.41
124	3	79.23	85.07	86.72	88.24	88.48	93.77	92.68	96.83	11.76
67	4	85.34	87.06	93.54	94.47	93.48	95.99	95.84	96.36	9.30
68	4	78.06	80.35	81.99	84.10	88.03	92.00	90.17	90.80	10.45
70	4	85.96	88.72	89.01	92.02	92.99	94.99	93.72	95.24	6.52
83	4	84.19	86.10	92.43	94.25	94.52	93.52	94.35	96.76	10.66
95	4	88.06	89.79	92.86	90.61	93.17	92.94	93.54	96.07	6.28
107	4	85.10	86.71	91.21	93.83	95.76	96.49	95.65	95.01	8.30
116	4	84.12	86.19	95.44	94.97	95.68	98.62	98.75	99.00	12.81
82	5	46.22	53.58	67.72	72.47	78.41	80.27	78.43	83.63	30.05
126	5	86.17	89.65	97.19	97.49	97.38	98.39	98.59	98.40	8.75

^1^ Feature dimensions; ^2^ experiment.
